# Pasteurized *Akkermansia muciniphila* Reduces Fat Accumulation via *nhr-49*-Mediated Nuclear Hormone Signaling Pathway in *Caenorhabditis elegans*

**DOI:** 10.3390/molecules27196159

**Published:** 2022-09-20

**Authors:** Zhongqin Wu, Yu Xiao, Fang Zhou, Jiaxu Chen, Xinming Chen, Aixiang Hou, Yuanliang Wang, Zongjun Li

**Affiliations:** 1 College of Food Science and Technology, Hunan Agricultural University, Changsha 410128, China; 2 Hunan Province Key Laboratory of Food Science and Biotechnology, Changsha 410128, China; 3 Key Laboratory of Ministry of Education for Tea Science, College of Horticulture, Hunan Agricultural University, Changsha 410128, China; 4 Longping Branch Graduate School, Hunan University, Changsha 526061, China

**Keywords:** *Akkermansia muciniphila*, lipid metabolism, *Caenorhabditis elegans*, fatty acid β-oxidation, fatty acid synthesis, *nhr-49* signaling pathway

## Abstract

Pasteurized *Akkermansia muciniphila* (p-AKK) is related to lipid metabolism and helps control obesity. The main goal of this study was to investigate the role and mechanism of p-AKK in lipid metabolism using *Caenorhabditis elegans*. The results showed that p-AKK increased the healthy lifespan of nematodes and helped maintain exercise ability in aging, suggesting a potential increase in energy expenditure. The overall fat deposition and triglyceride level were significantly decreased and the p-AKK anti-oxidative stress helped to regulate fatty acid composition. Additionally, the transcriptome results showed that p-AKK increased the expression of lipo-hydrolase and fatty acid β-oxidation-related genes, including *lipl-4*, *nhr-49*, *acs-2* and *acdh-8*, while it decreased the expression of fat synthesis-related genes, including *fat-7*, *elo-2* and *men-1*. These results partially explain the mechanisms underlying the fact that p-AKK decreases fat accumulation of *C. elegans* via *nhr-49*/*acs-2*-mediated signaling involved in fatty acid β-oxidation and synthesis.

## 1. Introduction

Obesity rates are increasing, affecting millions of people and their families annually [1]. It is a serious risk factor for various chronic diseases, such as hypertension, hyperlipidemia and type 2 diabetes [2,3,4]. Obesity is mainly characterized by an excessive increase in adipose tissue mass and dysregulation of lipid metabolism [5]. Thus, controlling obesity has attracted world-wide attention. Energy balance refers to the matching of energy intake and energy consumption, which plays an important role in lipid metabolism. When energy intake exceeds energy consumption, the energy balance is offset, resulting in an energy surplus, which leads to obesity. Triglycerides, the major type of fat stored in the adipose tissue, regulate energy expenditure and the fat metabolism balance [6]. People are now eating more and engaging in less physical activity; this leads to excess energy intake, which is the main cause of fat accumulation. 

Dietary interventions are effective ways of treating obesity and probiotics are widely reported to have an anti-obesity function. For example, it has been previously reported that probiotic supplements can influence systemic metabolism by affecting factors related to energy balance, such as intestinal barrier homeostasis, energy absorption and fat metabolism [7,8]. *Akkermansia muciniphila*, a new anaerobe probiotic, improves the intestinal niche and supports human health [9]. One study found that the oral administration of *A. muciniphila* to obese patients with insulin resistance improved lipid metabolism (triglyceride, cholesterol, etc.) without diet and exercise changes [10]. In another study, feeding high-fat-diet mice supplementation with *A. muciniphila* suppressed body weight gain, serum cholesterol levels and adipogenesis/lipogenesis, which prevented HFD-induced obesity [11]. Moreover *A. muciniphila* significantly lowered serum triglyceride via decreased expression of sterol-regulatory element binding protein (SREBP, regulator of triglyceride synthesis in liver tissue) in obese mice [12]. The above studies have confirmed that *A. muciniphila* can regulate fat metabolism and reduce fat accumulation. Moreover, its relationship with the host is reflected in the energy consumption associated with lipid metabolism [13,14]. Therefore, *A. muciniphila* has been increasingly studied as a potential anti-obesity microorganism.

The current research, however, has mostly focused on live bacteria instead of dead bacteria. It is reported that heat-killed bacteria retain key probiotic effects and the usage of these bacteria has more advantages over live probiotics mainly because of the safety profile [15]. In recent years, the probiotic effects of inactive bacteria have attracted widespread attention. One study found that heat-killed *Lactobacillus reuteri* GMNL-263 reduced liver fibrosis and the risk of nonalcoholic fatty liver disease by reducing transforming growth factor β expression levels [16]. Other studies have found that dietary heat-killed *Lactobacillus sakei* and *Lactobacillus gasseri* can regulate intestinal flora and provide immune protection in mice [17,18]. In particular, pasteurized *A. muciniphila* (p-AKK) increase energy expenditure and physical activity in mice on a high-fat diet, reducing obesity by promoting tissue thermogenesis [19,20,21]. It is suggested that heat-killed or alive AKK has a probiotic effect and can regulate energy metabolism to effect lipid metabolism. However, there is little evidence on the mechanism by which p-AKK regulates energy metabolism and on the relationship between *A. muciniphila* and host tissue fat metabolism.

*Caenorhabditis elegans* has been widely used as an in vivo model to explore the genetic regulation of fat storage due to its clear genetic information, conserved metabolic pathways and cheap and easy manipulation. There are four classic signaling pathways in the lipid metabolism of *C. elegans*: the nuclear hormone receptor *(nhr-49)* pathway, insulin/TGF-β, *sbp-1*/*mdt-15* pathway and amino-hexose pathways. Lin et al. found that *Momordica saponins* reduced fat stores via the *sbp-1/mdt-15* pathway [22]. Moreover, fermented barley β-glucan depends on *nhr-49* transcription factors and acts on the *nhr-49* pathway to reduce fat deposition in nematodes [23]. However, no relevant report has used nematodes to study the ability of p-AKK to prevent fat deposition. 

The present study aims to explore the protective effect of p-AKK bacteria, particularly on lipid metabolism, in a *C. elegans* model for the first time. The healthy lifespan and fat accumulation of nematodes fed with p-AKK were measured. The effect of p-AKK on the metabolism of nematodes was further analyzed using transcriptome sequencing. This study provides an important theoretical basis for the safe application of *A. muciniphila* to food or health products.

## 2. Materials and Methods

### 2.1. Reagents and Strains

*A. muciniphila* (ATCC BAA-835), purchased from Beijing Biobw Biotechnology Co., Ltd. (Beijing, China), was cultured in sterilized brain heart infusion broth at 37 °C in an airtight pot with an anaerobic workstation for approximately 48 h to reach a late exponential growth phase under strict anaerobic conditions. *Escherichia coli* OP50 was cultured in sterilized terrific broth at 37 °C in a constant-temperature shaker incubator for 12 h. The two bacteria were then centrifuged at 8000 rpm for 10 min and washed with sterile water twice. Then, the bacterial cells were re-suspended with 100 mg/mL sterile M9 buffer (wet weight). Finally, heat treatment took place at 70 °C in an electric-heated thermostatic water bath for 30 min of fresh *A.muciniphila* and *E. coli* OP50. They were then immediately stored at −20 °C until use.

*C. elegans* and *E. coli* OP50 were originally obtained from Caenorhabditis Genetics Center (University of Minnesota, MN, USA). N2 Bristol was used as the wild-type strain. *C. elegans* were washed off the plates into 10-mL centrifuge tubes and lysed with a bleaching mixture (5 mL 1 M NaOH, 1.5 mL 6% NaClO, 3.5 mL ddH_2_O). Age-synchronized populations of L1-larval nematodes were obtained as described previously [24]. Worms were maintained and propagated in peptone-free modified nematode growth medium (mNGM) at 20 °C using standard techniques. Worms were divided into the normal group treated with live OP50, the p-OP50 group treated with pasteurized OP50 and the p-AKK group treated with p-AKK. *C. elegans* (N2) fed normally until to the larval L4 stage and they were then treated with different bacteria for 24 h. 

### 2.2. Lifespan Assay

*C. elegans* were cultured on fresh mNGM plates without starvation and lifespan analysis was conducted at 20 °C unless otherwise stated. Late L4 larvae were transferred to mNGM plates containing live OP50 (normal group), p-OP50, or p-AKK and 0.12 mM FUdR to inhibit progeny growth. The day that L4 larvae or young adults were transferred to an mNGM plate was defined as test day 0. Then, the worms were transferred to fresh plates with different bacteria every day until all nematodes were dead. The worms that did not respond to a mechanical stimulus were scored as dead. The experiments were independently repeated three times. Kaplan-Meier analysis was used to analyze survival after treatments.

### 2.3. Healthy Lifespan: Body Bends/Movements on mNGM Plates

The L4 late larvae were transplanted onto experimental plates and cultured for 24 h. At that time, nematodes were 4 d old. The body bends and movements of the 4-, 6-, 8-, 10- and 12-d-old nematodes were counted. 

The body movement frequency (normal locomotion) in each nematode was observed and recorded at intervals of 30 s under a stereomicroscope (Nikon Eclipse E100) equipped with TCapture Application video-recording software (version 3.9.0.604, Nikon Instruments, Melville, NY, USA). There were 10 worms in each group and the experiments were independently repeated three times. The body movement frequency was defined as the number of sine waves in 30s [25,26].

In brief, worms were classified as class ‘‘A’’ when they showed spontaneous movement or vigorous locomotion in response to prodding, class ‘‘B’’ when they did not move unless prodded or appeared to have uncoordinated movement and class ‘‘C’’ when they moved only their head and/or tail in response to prodding. Dead worms were classified as class ‘‘D’’ [27]. 

### 2.4. ATP Quantification Assay

Worms in each group were washed from the plates with M9 buffer and transferred to fresh tubes. Then, the homogenates of worms were prepared using an ultrasonic cell crusher. After centrifuging at 8000× *g* for 10 min, the supernatants were used for the next assay. 

ATP levels were measured as described [28] and per the instructions of an ATP-detection kit (Shanghai ZCIBIO Technology Co., Ltd., Shanghai, China). The protein concentration was quantified using a BCA protein assay kit (Nanjing Jiancheng Bioengineering Institute, Nanjing, China).

### 2.5. Triglyceride Quantification

Triglyceride quantification in *C. elegans* was conducted with a commercial TG assay kit (Nanjing Jiancheng Bioengineering Institute, Nanjing, China). Protein content was used as the internal control to normalize the triglyceride level.

### 2.6. Oil Red O Staining

An Oil Red O staining assay was performed according to the previous protocol [29]. After collecting nematodes with M9 buffer, we added Oil Red O working solution and stained them for 2 h. Then, we removed the excess dye by centrifugation and resuspended them with M9 buffer. Finally, 5 µL of worm solution was taken on 2% agarose, observed under a microscope and photographed. Images were analyzed for color density using Image J software.

### 2.7. Measurement of Reactive Oxygen Species (ROS)

The measurement of ROS scavenging assay was performed in accordance with the literature method with slight modifications [30]. The treated nematodes were washed twice with M9 buffer to remove bacterial interference. The worms were then transferred and the worm concentration was adjusted to 1000 worms/mL with M9 buffer containing 5 µM H_2_DCFDA (Sigma-Aldrich.Louis, MO, USA). They were pre-incubated for 3 h at 20 °C. Then, 50 µL per well of M9 buffer with probe and nematodes were loaded in a 96-well plate and read with a fluorescent microplate reader in a microplate photometer (Thermo Scientific™ Multiskan™ FC, Waltham, MA, USA) for fluorescence quantification at an excitation wavelength of 485 nm and an emission wavelength of 530 nm every 10 min for a total time of 2 h. The quantified ROS was expressed as increase in fluorescence in 2 h.

### 2.8. Antioxidant Enzyme Assay

Catalase (CAT), glutathione peroxidase (GSH-PX) and superoxide dismutase (SOD) activities were determined using Solarbio kits (Solarbio Science and Technology Co.,Ltd., Beijing, China) per the manufacturer’s instructions. The BCA protein assay kit was used to quantify the protein concentrations of the worms’ homogenates.

### 2.9. Transcriptome Sequencing

Total RNA was extracted using Trizol reagent at 4 °C and the quality was assessed in a Nanodrop2000 and checked using RNase-free agarose gel electrophoresis. The mRNA was isolated and enriched using oligo(dT) magnetic beads. First-strand cDNA was generated using random hexamer-primer reverse transcription, followed by synthesis of the second-strand cDNA using RNase H and DNA polymerase I. Then, single-end and paired-end RNA-Seq libraries were prepared following Illumina’s protocols and sequenced on the Illumina NovaSeq 6000 by Shanghai Majorbio Biopharm Technology Co., Ltd. (Shanghai, China).

Reads obtained from the sequencing machines needed to have the clean reads filtered, aligned with ribosome RNA, aligned with the reference genome and the gene abundance quantified. Finally, RNA differential expression analysis was performed using DESeq2 software between two different groups and edgeR between two samples. The genes/transcripts with a false discovery rate (FDR) below 0.05 and absolute fold change ≥ 2 were considered differentially expressed genes (DEGs)/transcripts. 

All DEGs were mapped to GO terms in the Gene Ontology database (http://www.geneontology.org/, accessed on 7 January 2022). Kyoto Encyclopedia of Genes and Genomes (KEGG) pathway enrichment analyses were used to identify significant pathways. The calculated p-value underwent FDR correction, with FDR ≤ 0.05 as a threshold.

### 2.10. Lipid Extraction and Gas Chromatography/Mass Spectrometry (GC-MS) Assays

Total fat was extracted and methylated as previously described, with slight modifications [31,32]. Several thousand adult worms were washed into a screw-cap glass bottle and centrifuged at 1000× *g* for 1 min to remove as much water as possible. Then, 1 mL of 2.5% H_2_SO_4_ in methanol was used to extract fatty acids from tissues and trans methylate them. The sample was capped and incubated at 80 °C for 1 h. After the addition of 0.3 mL of hexane and 1.5 mL of H_2_O, the fatty acid methyl esters were extracted into the hexane layer by shaking and centrifuging the tubes at low speed.

Fatty acid methyl esters were then injected for GC-MS analysis using an HP-5MS column (30 m × 0.25 mm × 0.25 µm) with helium as the carrier gas. The temperature of the injector was 250 °C. The running condition was as follows: the initial temperature of 60 °C was increased to 150 °C by 15 °C/min, held for 3 min, increased to 195 °C by 5 °C/min, held for 5 min, increased to 230 °C by 6 °C/min, held for 3min and increased to 260 °C by 3 °C/min. The desaturation index was calculated as the ratio of C18:1/C18:0. 

### 2.11. qRT-PCR

Approximately 2000 synchronized late L4 larvae were transferred to mNGM plates containing live OP50, p-OP50, or p-AKK and cultured at 20 °C for 24 h. Total RNA was extracted using Trizol reagent and converted to cDNA using a HiScript ll Q RT SuperMix Kit (Enzyme Biotech Co., Ltd., Beijing, China). The qRT-PCR reactions were performed using Power SYBR Green PCR Master Mix (Vazyme Biotech Co., Ltd., Nanjing, China) and the Rotor-Gene Q system. The relative expression levels of the genes were evaluated using the 2^−ΔΔCT^ method and normalized to the expression of the *β-actin* gene [33]. The expression of the *lipl-4, acs-2, ech-1.1, cpt-4, fat-5, fat-6, fat-7, nhr-49, sbp-1 and mdt-15* genes was determined and the primers used here are listed in Supplementary Material S1.

### 2.12. Statistical Analysis

Statistical analysis of the fatty acid and qRT-PCR analyses was performed using an independent sample t-test. Statistically significant differences and extremely significant differences were defined as (*) *p* < 0.05 and (**) *p* < 0.01 compared to the normal group, respectively. The results of other analyses were analyzed using a one-way analysis of variance (ANOVA) with Duncan’s tests for post hoc comparison using IBM SPSS Statistic 25.0 software. The data were presented as the arithmetic mean ± standard deviation (SD). The different letters above the columns denote significant differences (*p* < 0.05). The diagrams were created using Graph Prism 6.0.1 for Windows. mRNA-seq analysis was performed using the Majorbio Cloud Platform (www.majorbio.com, accessed on 7 January 2022).

## 3. Results

### 3.1. Effect of p-AKK on Healthy Lifespan in C. elegans

**Figure 1:**
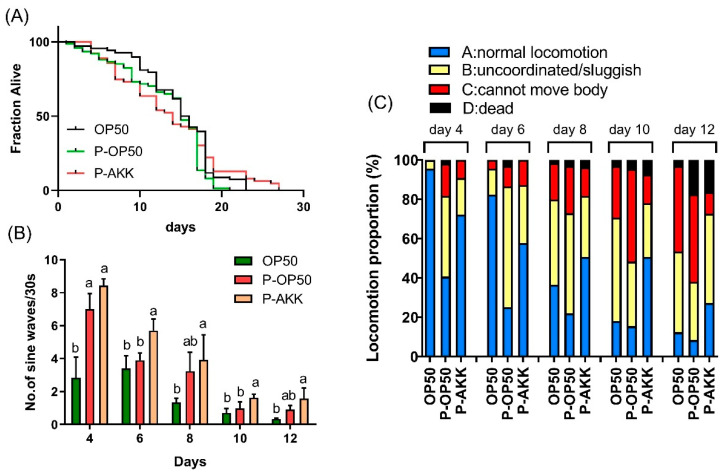
Positive effect of p-AKK on nematodes’ healthy lifespan. (**A**) Analysis of lifespan of nematodes fed with different bacteria. N = 3; (**B**) Analysis of normal locomotion frequency with increasing age of nematodes fed with different bacteria. There were 10 worms in each group and the experiments were independently repeated three times; (**C**) Analysis of changes in movement state with increasing age of nematodes fed with different bacteria. The results are expressed as the mean ± SD and *p*-values were calculated by Duncan’s tests. Significant differences between groups are expressed by different letters (a,b) within the same day (*p* < 0.05). N = 3.

To explore whether p-AKK has health benefits of *C. elegans*, we evaluated the effects of p-AKK on lifespan and age-related physiological functions, including motricity. p-AKK had no significant effect on the lifespan of nematodes, with an average lifespan of 14 d, which is like that of normal nematodes (Figure 1A). However, p-AKK had a certain effect on the longest lifespan of nematodes, which reached 27 d, while the longest lifespan of p-OP50 group nematodes as the control was only 21 d (Table 1). In terms of healthy lifespan, the body bends and movement of nematodes were used as health evaluation indicators during the nematodes’ aging process. We analyzed the motility status of 4-, 6-, 8-, 10- and 12-d-old nematodes grown on live OP50, p-OP50, or p-AKK diets. *C. elegans’* locomotion activity declined with age on both control and heat-killed bacteria diets (Figure 1B,C). By 8 d of age, the movement speed of nematodes in the normal group decreased to half of the original state and only 36% of the nematodes maintained sinusoidal wavelength movement. While 50% of the nematodes in the p-AKK group maintained sinusoidal wavelength movement, the movement speed was significantly higher than that in the normal group and the p-OP50 group (*p* < 0.05). At the age of 12 day, the normally reared nematodes appeared aged and most of the nematodes could not move normally, but the p-AKK group nematodes had a higher coordinated movement ratio and speed. Therefore, these data show that p-AKK has a positive effect on the healthy lifespan of nematodes and can delay the loss of motor activity during aging.

### 3.2. Effect of p-AKK on Energy Metabolism

**Figure 2:**
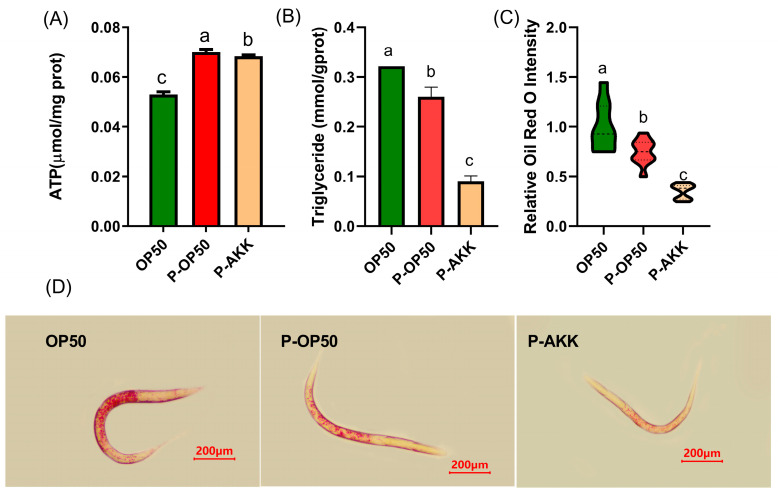
p-AKK enhances cellular energy currency and reduces fat accumulation. N = 3. (**A**) ATP level of *C. elegans*. (**B**) Triglyceride content of *C. elegans*. N = 3. (**C**) Quantification of data shown in (**D**). N = 10. (**D**) Oil Red O staining of fixed worms and selected intuitive characteristic map. Bar, 200 μm. The results are expressed as the mean ± SD and *p*-values were calculated by Duncan’s tests. Significant differences between groups are expressed by different letters (a–c) (*p* < 0.05).

The ATP content of the p-AKK and p-OP50 groups was significantly higher than that of the normal group, which suggests that p-AKK enhanced the energy currency (Figure 2A). The higher energy currency indicates that the metabolic activity of nematodes is stronger, which reduces the excess energy metabolism and avoids fat deposition. *C. elegans* lacks the dedicated adipocytes present in mammals, but this organism stores fat in droplets in its intestinal and hypodermal cells. To assess the effects of p-AKK on the overall storage of lipid in *C. elegans*, we examined the triglyceride level and stained nematodes with Oil Red O. The triglyceride level was decreased by feeding with p-AKK; that is, it was 35% lower than that of those fed p-OP50 (Figure 2B). The Oil Red O staining results showed that less fat in the p-AKK group compared the OP50 and p-OP50 group, which suggested that p-AKK intake resulted in lower fat accumulation in *C. elegans* (Figure 2C,D). That is consistent with the triglyceride results. Therefore, these data suggest that p-AKK may play an important role in increasing energy currency and reducing fat storage in *C. elegans*.

### 3.3. Effect of p-AKK on Antioxidant Capacity in C. elegans

**Figure 3:**
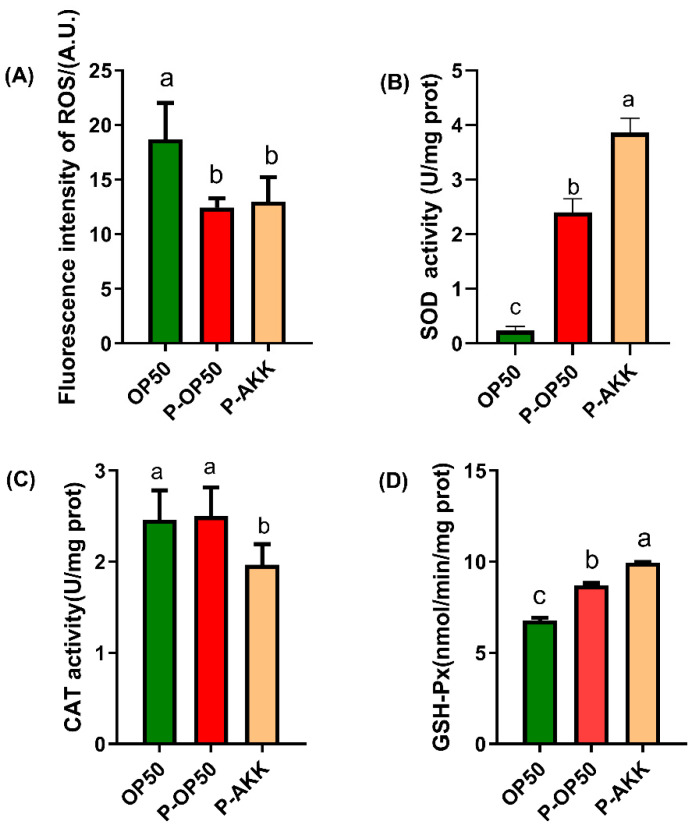
Increase in antioxidant capacity and decrease in accumulation of ROS in *C. elegans* after p-AKK treatment. (**A**) Results of ROS labeling and detection after treating L4 nematodes with different bacteria for 24 h. N = 3. (**B**–**D**) SOD, CAT and GSH-PX content in L4 nematodes after feeding with different bacteria for 24 h. N = 3. The results are expressed as the mean ± SD and *p*-values were calculated by Duncan’s tests. Significant differences between groups are expressed by different letters (a–c) (*p* < 0.05).

ROS accumulation level and antioxidant enzyme activity were assessed. As shown in Figure 3A, the ROS level of the p-AKK and p-OP50 group was significantly lower than that of the normal group (*p* < 0.05). SOD and GSH-PX were enhanced in the p-AKK group (60.90% and 14.39% higher than those of the p-OP50 group, respectively). However, p-AKK did not increase the CAT activity in *C. elegans*. These results suggest that p-AKK could enhance the ability of ROS scavenging through increased SOD and GSH-PX activity in worms.

### 3.4. Effect of p-AKK on Fatty Acid Composition in C. elegans

**Figure 4:**
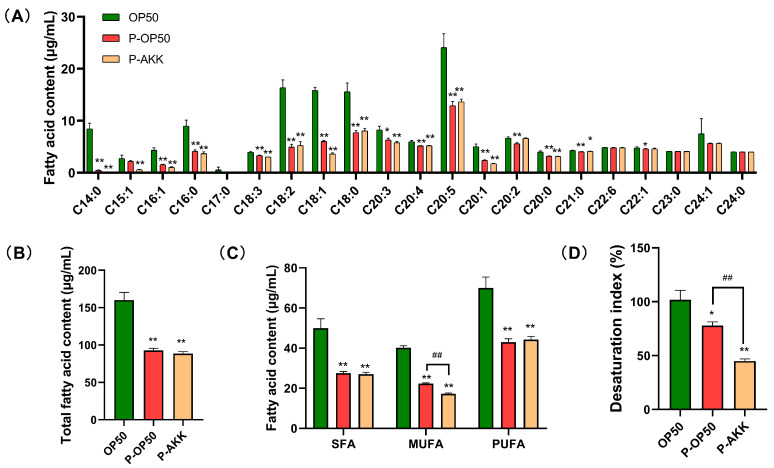
Decrease in the proportion of MUFAs by p-AKK treatment. (**A**) Total fatty acid profiles of *C. elegans* treated with different bacteria analyzed using GC-MS (N = 3). The contents of each fatty acid were calculated. (**B**) The contents of total fatty acids in each group of nematodes were calculated (N = 3). (**C**) SFA, MUFA and PUFA levels in worms fed different bacteria (N = 3). (**D**) The desaturation index of fatty acid in each group were calculated (N = 3). Error bars represent SD. Using an independent sample *t*-test, statistically significant differences and extremely significant differences were defined as (*) *p* < 0.05 and (**) *p* < 0.01 compared to the OP50 group, respectively. (^##^) *p* < 0.01 compared to the p-OP50 group.

The fatty acid composition was analyzed using GC-MS in nematodes. In the p-AKK group, the overall fatty acid levels measured decreased significantly and the ratio of composition changed (Figure 4A,B). Palmitoleic acid (C16:1) and oleic acid (C18:1) are two important substrates of triglyceride biosynthesis. p-AKK significantly reduced their content, which was consistent with the triglyceride content detection results. The levels of monounsaturated fatty acids (MUFAs), such as C15:1, C16:1, C18:1 and C20:1, were lower in the p-AKK group than in the other two groups (Figure 4C). However, the saturated fatty acid (SFA) and polyunsaturated fatty acid (PUFA) expression patterns were similar in the p-AKK and p-OP50 groups, showing dynamic down regulation. Moreover, the desaturation index of fatty acids was 32.93% lower in the p-AKK group than in the p-OP50 group (*p* < 0.01) (Figure 4D). These results suggest that p-AKK-reduced fat accumulation may be related to the downregulation of the MUFA proportion in *C. elegans*.

### 3.5. p-AKK Upregulates Energy Metabolic Pathways

To further reveal the potential mechanism by which p-AKK reduces lipid accumulation via increased energy metabolism in nematodes, RNA-Seq was used to determine the alternation of biological processes and the expression of key genes by p-AKK in nematodes.

#### 3.5.1. Identification of DEGs

**Figure 5:**
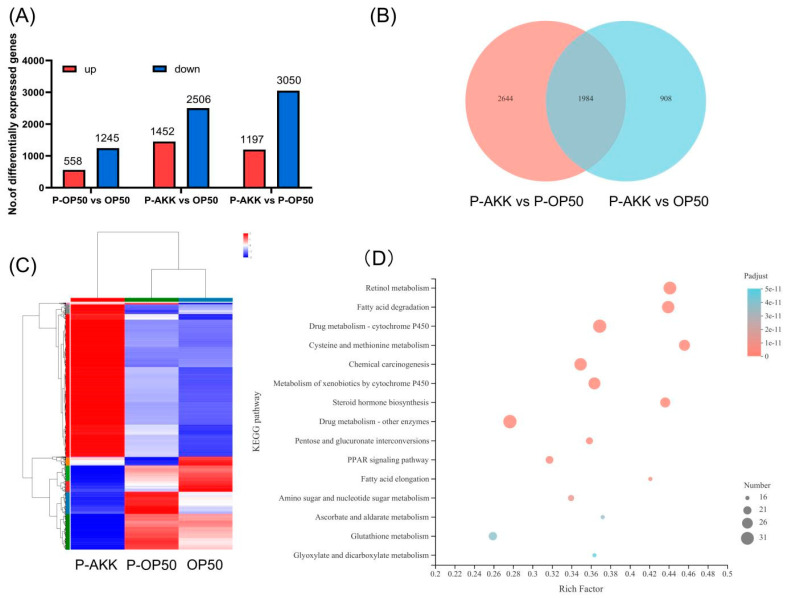
Changes in gene expression profiles of worms fed different bacteria affect energy metabolism. (**A**) Numbers of DEGs in pairwise comparisons of three treatments. (**B**) Venn diagram showing the number of DEGs in different treatments. (**C**) Heatmap diagrams showing relative expression levels of total DEGs among three treatments. (**D**) Top 15 statistics of KEGG pathway enrichment analysis of worms fed p-AKK and p-OP50.

A rigorous comparison at adjusted *p* ≤ 0.05 and log2FC fold change ≥ 2 was performed to identify the number of DEGs for different groups. In total, the gene expression analysis showed that 3958 genes were significantly differentially expressed, including 2506 upregulated and 1452 downregulated genes in the p-AKK group compared with the normal group. Of a total of 3247 DEGs in *C. elegans*, 2050 genes were upregulated and 1197 genes were downregulated under p-AKK diets compared with p-OP50 diets (Figure 5A). In the two comparisons, 1984 DEGs were counted (Figure 5B). IPath 3.0 software (version 3, https://pathways.embl.de/, accessed on 7 January 2022) was used to visually analyze the metabolic pathways involved in these DEGs and the metabolic information of the whole biological system was apparent. Most of the DEGs were enriched in energy metabolism (Supplementary Material S2), which was consistent with previous detection indicators. Next, a general overview of the expression pattern was visualized in a heatmap, which provided an overall understanding of the changes in gene expression. The *C. elegans* fed p-OP50 and those fed live OP50 showed similar gene expression patterns, while those fed p-AKK and p-OP50 exhibited opposite gene expression patterns (Figure 5C).

Therefore, to explore the metabolic changes in nematodes fed p-AKK, we chose the p-OP50 group as the control group for further comparison. In GO and KEGG functional analyses, we focused on the differential genes related to energy metabolism induced by *A. muciniphila*.

#### 3.5.2. Functional- and Pathway-Enrichment Analyses of DEGs Induced by p-AKK

Based on GO and KEGG pathway annotation analysis (Supplementary Material S3), identified gene targets were functionally related to energy-related terms (such as transport and catabolism) and lipid metabolism pathways. Therefore, the dietary regulation of p-AKK may have a significant effect on lipid metabolism. Figure 5D shows the top 15 ranked KEGG pathway terms of DEGs. Generally, the larger the enrichment factor, the more significant the influence. The pathways with enrichment factors above 0.4 were retinol metabolism, fatty acid degradation, cysteine and methionine metabolism, steroid biosynthesis and fatty acid prolongation. These enrichment pathways are closely related to energy metabolism, especially fatty acid metabolism. These results could provide essential information on the effect of p-AKK on fatty acid metabolism.

#### 3.5.3. Identification of DEGs Induced by p-AKK in *C. elegans*

Fat metabolism is an important source of energy and this metabolic pathway is highly conserved in organisms. Therefore, we looked further at the differential metabolism genes related to fatty acid metabolism to understand which metabolic enzymes are affected (see Supplementary Material S4). In the fatty acid degradation pathway, the following related genes were significantly upregulated: *lipl-4* (encoding lipase), *acs-1* and *acs-2* (encoding fatty acid CoA synthetase), *cpt-4* (encoding carnitine palmitoyl-transferase I) and *acdh-8* (encoding acetyl-CoA dehydrogenase). Significantly downregulated genes included *acox-1.2/-3* (encoding acetyl-CoA oxidase) and *ech-1.1/-6/-7/-9* (encoding hydratase). In the PPAR signaling pathway, the expression of *men-1* (encoding malate dehydrogenase) and *fat-7* (encoding delta (9)-fatty acid desaturase) was downregulated. In the fatty acid prolongation pathway, *acaa-2* (encoding acetyl-CoA transferase), *elo-2/-3/-5/-9* (encoding long-chain fatty acid elongation protein) and *art-1* (encoding very-long-chain enoyl-CoA reductase) were downregulated, while *ppt-1* (encoding palmitoyl-protein thio-esterase 1) was upregulated. An analysis of the functions of the above differential genes suggested that p-AKK can release energy by promoting lipid hydrolysis and fatty acid β-oxidation while inhibiting fatty acid synthesis to reduce fat accumulation. Finally, it affects the energy currency of nematodes, providing sufficient energy to balance the energy consumption caused by exercise.

### 3.6. qRT-PCR Validation of DEGs

To further verify the accuracy of transcriptomic data and the possible mechanism of p-AKK affecting lipid metabolism in nematodes, we selected five genes related to lipid degradation for qRT-PCR validation. Supplementary Material S5 confirmed that the expression patterns of these genes were very similar to the transcriptome sequencing data. Therefore, the mRNA-Seq sequencing data are reliable and consistent with our hypothesis.

**Figure 6:**
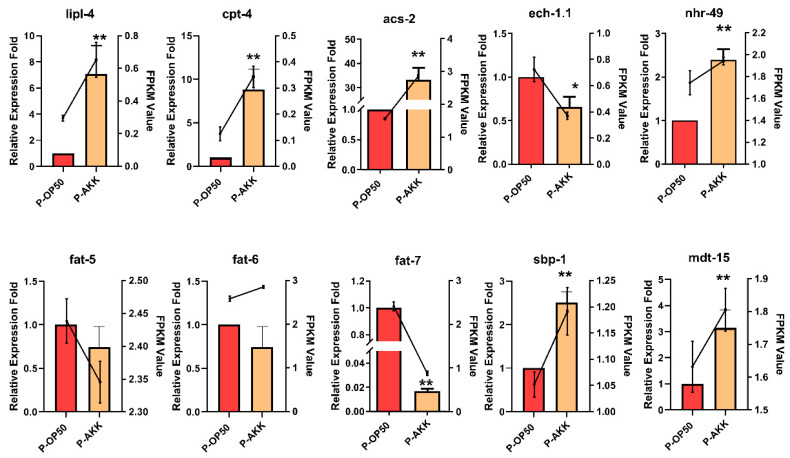
qRT-PCR analysis of expression of 10 DEGs. Line charts are from the transcriptome data results (FPKM value). Column charts are from the qRT-PCR results (relative expression fold change). Bars represent means ± SD (N = 3). Using an independent sample *t*-test, statistically significant differences and extremely significant differences were defined as (*) *p* < 0.05 and (**) *p* < 0.01 compared to the P-OP50 group, respectively.

Further analysis revealed that *nhr-49* is functionally like mammalian PPAR, which is necessary for fatty acid β-oxidation, fatty acid-binding and fatty acid desaturation. In mammals and nematodes, *nhr-49*/*mdt-15* has a similar function, as it regulates the energy balance and part of the metabolic genes [34,35]. *Sbp-1* is a coactivating transcription factor of *mdt-15*, a homolog of the mammalian sterol regulatory element-binding protein (SREBP). As the upstream regulator of stearoyl-CoA desaturase (SCD), it is responsible for the rate-limiting link of catalyzing saturated fatty acids to monounsaturated fatty acids. In nematodes, SCD is encoded by *fat-5, fat-6* and *fat-7*. Therefore, we analyzed the expression of the above genes. Figure 6 shows that p-AKK significantly upregulated the expression of *acs-2, lipl-4, cpt-4, nhr-49, sbp-1 and mdt-15* and it upregulated the expression of the *acs-2* gene by nearly 30-fold. However, p-AKK significantly downregulated the expression of *fat-7* and *ech-1.1*. The above genes are involved in lipid hydrolysis, fatty acid β-oxidation and fatty acid synthesis. These results suggest that p-AKK upregulates the *nhr-49* pathway to enhance fatty acid β-oxidation and prevent fat accumulation in nematodes. 

## 4. Discussion

Increasing evidence shows that p-AKK offers unique health benefits for various problems, such as obesity, insulin resistance and glucose tolerance [36]. However, due to the disadvantages of current models, it has been difficult to conduct in-depth research. Therefore, the mechanism by which p-AKK regulates obesity and reduces the risk of metabolic diseases remains unclear. Unlike these models, a nematode model may be more appropriate to explore dietary intervention and the indirect intake of p-AKK in vivo-mediated regulation of fat metabolism and its exact mechanism.

This study investigated the effect of dietary intake of p-AKK on lifespan and energy metabolism in *C. elegans*. p-AKK contributes to a healthy lifespan in nematodes, improves motor activity during aging and enhances thermogenic effects. This is consistent with previous studies in rodent models [21]. The increase in heat production and energy consumption is often accompanied by an acceleration in metabolism. Previous studies have reported that the intestinal flora contributes to the absorption of carbohydrates and lipids and confirmed the crucial roles of *A. muciniphila* in energy metabolism [37]. Moreover, in one study, p-AKK increased the energy in mouse feces, indicating a decrease in energy absorption in the body, which may explain the decrease in weight gain [36]. In our study, p-AKK increased the level of ATP in nematodes but significantly decreased fat storage. This may be because p-AKK reduces the energy absorption of nematodes and converts energy into heat for the energy consumption of exercise. 

An excess in the availability of nutrients can lead to mitochondrial dysfunction and even increased ROS production. However, these changes were associated with alterations in adipogenesis, lipolysis and fatty acid esterification [38]. The adipokines generated from adipose tissues induce the production of ROS, which generates oxidative stress [39]. Antioxidant enzymes protect organisms against oxidative stress. If a large amount of ROS is produced and it overcomes the antioxidant defense, it damages DNA and membrane lipids, which induces the lipid peroxidation of polyunsaturated fatty acids [40] and fat accumulation [41]. In the current study, p-AKK decreased ROS content to resist oxidative stress in *C. elegans* and this protective effect was related to enhancing SOD and GSH-PX content in *C. elegans*. p-AKK protected nematodes from oxidative stress and avoided PUFA peroxidation. Although the fatty acid ratio showed a dynamic balance, it significantly reduced the total fatty acid content in nematodes. This suggests that p-AKK resistance to oxidative stress can assist nematodes in reducing fat storage in healthy status. Although p-OP50 has a similar impact in these aspects as p-AKK, the effect of p-AKK was more pronounced, perhaps due to the difference in bacterial components.

Based on the above results, we further comprehensively studied the effect of p-AKK on the energy metabolism, particularly lipid metabolism, of *C. elegans.* The possible mechanism is further explored using transcriptome data. Changes in diet can have a profound impact on gene expression, particularly those that encode metabolic enzymes [42]. RNA-Seq data show that an increase in the level of genes involved in fat metabolism, particularly some metabolic enzymes, affects the balance of energy conversion. The *lipl-4* gene is related to the hydrolytic activity of lipase and its upregulated expression can accelerate lipid hydrolysis and release free fatty acids. Fatty acid degradation requires the activation of fatty acyl-CoA synthase (*acs-2* gene-encoded) before it can enter the mitochondria for β-oxidative decomposition under the transport of carnitine palmitoyl-transferase I (CPT-4) [43]. Thus, *acs-2* is the key catalysis enzyme for fatty acids to enter β-oxidation and the upregulation of its expression level indicates the enhancement of fatty acid oxidation. 

In this study, p-AKK increased the transcription of *lipl-4, cpt-4 and acs-2* and enhanced the biological process of fatty acid β-oxidation. *nhr-49* is a key transcription factor for regulation of β-oxidation and the peroxisome pathway, which is the regulatory center of lipid metabolism [44]. *ech-1.1* genes encode mitochondrial hydratase, which participates in the β-oxidation of lipids [45]. In addition, *ech-1.1* and *acs-2* are both downstream target genes of *nhr-49* that positively regulate lipolysis [46]. *mdt-15* specifically binds to *nhr-49* and is positively correlated with *acs-2* [35,47]. p-AKK increased the expression of *nhr-49* and *mdt-15* genes, particularly the *acs-2* gene, but inhibited the transcriptional level of *ech-1.1*. The overexpression of *acs-2* makes nematodes show a low-fat phenotype, which indicates that it is a key target for p-AKK to regulate lipid metabolism. Meanwhile, several studies have reported that natural products can reduce fat by upregulating the *acs-2* gene, providing support for the above hypothesis [48]. 

*Sbp-1* plays an important role in overall fat synthesis. *Sbp-1*, as a transcriptional coregulator of *mdt-15*, positively feeds back to δ9 fatty acid desaturase (SCD-1), which is a signal target for regulating lipid synthesis [22]. The results indicated that p-AKK upregulated the expression of the *sbp-1* gene, but had no positive effect on SCD-1 enzyme. This situation maybe that *sbp-1* is affected by other lipid metabolism genes, making it counteract to execute feedback commands. Besides, p-AKK inhibited the expression of *fat-7* (encodes SCD-1) and altered stearic to oleic acid ratios, resulting in a decrease in the content of total fatty acids in nematodes. This suggests that inhibiting the expression level of SCD-1 may help reduce overall fat storage. Moreover, Marc R. found that RNAi of *fat-7* causing reduced fat content and increased expression of genes in β-oxidation, including *acs-2* [49]. This evidence supports our results and demonstrating that *fat-7* can regulates β-oxidation through an apparent negative feedback mechanism. This mechanism is similar to that of leptin downregulating SCD-1 and increasing lipid oxidation to express uncoupling proteins and induce the body to burn excess calories [50].

In summary, this study proposed that, through dietary intake, p-AKK activates the *nhr-49* pathway, enhances fatty acid transport and β-oxidation and negatively feeds back to SCD-1 to reduce fat production and keep nematodes in a low-energy state to improve health. However, due to resource limitations, we did not carry out target verification on nematode mutants. Nevertheless, this study’s experimental results provided the relationship between thermal p-AKK and health status and supplemented the basic evidence on p-AKK’s regulation of energy metabolism.

## 5. Conclusions

**Figure 7:**
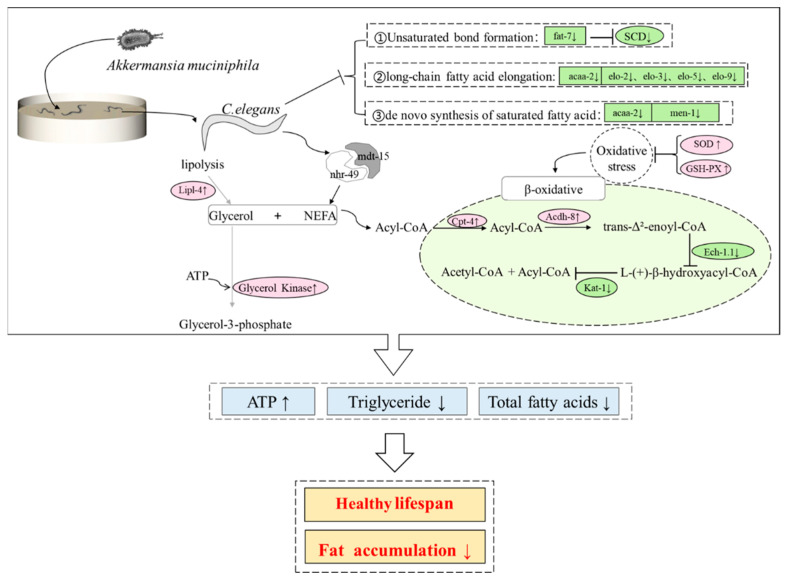
The molecular mechanism of p-AKK reduces fat accumulation.

p-AKK can improve spontaneous exercise ability, support a healthy lifespan, enhance antioxidant capacity and reduce triglyceride content in *C. elegans*. Oil Red O staining also confirmed the effect of p-AKK on fat accumulation in *C. elegans*. These results, along with those of the RNA-seq and qRT-PCR analyses, suggest that the molecular mechanism of p-AKK decreased fat accumulation in *C. elegans* may be that *lipl-4* participates in fat hydrolysis by upregulating expression, which can hydrolyze fat into fatty acids and glycerol. Fatty acids may be activated by the action of acyl-CoA synthase, which catalyzes the formation of fatty acyl-CoA. Then, it would enter the mitochondria for β-oxidation under the transport of carnitine palmitoyl-transferase I. Under the catalysis of acyl-CoA dehydrogenase, it was dehydrogenated to trans-double-bond fatty acyl-CoA and promoted the hydration process. However, p-AKK may inhibit the activity of hydratase, resulting in an abundance of fatty acyl-CoA dehydrogenase. Thus, the expression of *acs-2* was significantly upregulated. Furthermore, p-AKK upregulates expression of *nhr-49* and *mdt-15* which, as upstream genes of *acs-2*, further accelerates the accumulation of *acs-2*. Meanwhile, p-AKK inhibits lipid biosynthesis by inhibiting the expression of long-chain fatty acid elongation proteins, acetyl-CoA transferase, malate dehydrogenase and δ9 fatty acid desaturase (Figure 7). Therefore, p-AKK may accelerate fatty acid β-oxidation and inhibit lipid synthesis by activating the *nhr-49* pathway to balance the increased energy consumption of exercise and reduce fat accumulation *of C. elegans*. 

## Figures and Tables

**Table 1:** Lifespans of nematodes treated with different types of bacteria.

Group	Number of Worms	Mean Lifespan (Days)	Maximum Lifespan (Days)	Median (Days)
Alive OP50	68	14.88 ± 0.57	23.00	15.00 ± 0.63
Pasteurized OP50	74	13.43 ± 0.58	21.00	15.00 ± 0.57
Pasteurized AKK	66	13.87 ± 0.80	27.00	14.00 ± 1.31

## Data Availability

The datasets used and analyzed during the current study are available from the corresponding author on reasonable request.

## Supplementary Materials

The following supporting information can be downloaded at: https://www.mdpi.com/article/10.3390/molecules27196159/s1. Supplementary Material S1. Primer sequences; Supplementary Material S2. IPath 3.0 visually analyze the metabolic pathways of nematodes fed with different bacteria; Supplementary Material S3. GO and KEGG analysis of DEGs between worms fed p-AKK and p-OP50; Supplementary Material S4. KEGG Pathway; Supplementary Material S5. DEGs and mRNA levels in fat metabolism of worms treated with p-AKK and p-OP50.
