# Supplementary material for: Pasteurized Akkermansia muciniphila Reduces Fat Accumulation via nhr-49-Mediated Nuclear Hormone Signaling Pathway in Caenorhabditis elegans
Source: Molecules. 2022 Sep 20;27(19):6159. doi: 10.3390/molecules27196159 (PMC9572206; doi:10.3390/molecules27196159)
Supplement: Supplementary file 1 [file molecules-27-06159-s001.zip › molecules-1870823-supplementary.pdf]

Supplementary Material S1: Primer sequences

Table S1. Primer sequences.

Genes	Forward primer sequence (5' to 3')	Reverse primer sequence (5' to 3')
β-actin	GCCGGAGACGACGCTCCACGCG	GCCTCGTCTCCGACGTACGAGTC
lipl-4	ATGGCCGAGAAGTTCCTACATCGT	GGTGAATTGGCGACCCAATCGAAA
acs-2	GCAGCCTCGCTCTACACTCT	GACTCCTGCAAATGCACATGC
ech-1.1	CGTCGGAGCTGGATTCATGG	TCGTTCAACGCCTGCCTGGT
fat-5	CGGCCGCCCTCTTCCGTTAC	TGGCTGCCATCCGACCCAGT
fat-6	TCAACAGCGCTGCTCACTAT	TTCGACTGGGGTAATTGAGG
fat-7	CAACAGCGCTGCTCACTATT	CACCAACGGCTACAACTGTG
cpt-4	TGACAATCGCACCTCCACAG	TGAATGCTTCGTCCCTCGTT
nhr-49	AGGCTCGTGTCAATCAAGAGATGTG	ATGCCGATGCTCCAGAATCACTTC
sbp-1	CACCACCTCATCACCACCATCAC	TTCGTCTCTGGAGCATCTTCAATCG
mdt-15	CAGAGACTTGAGCCTGAGTTGGCG	GTCCTTCGACATACACTTTGCGAAC

Supplementary Material S2: IPath 3.0 visually analyze the metabolic pathways of nematodes fed with different bacteria

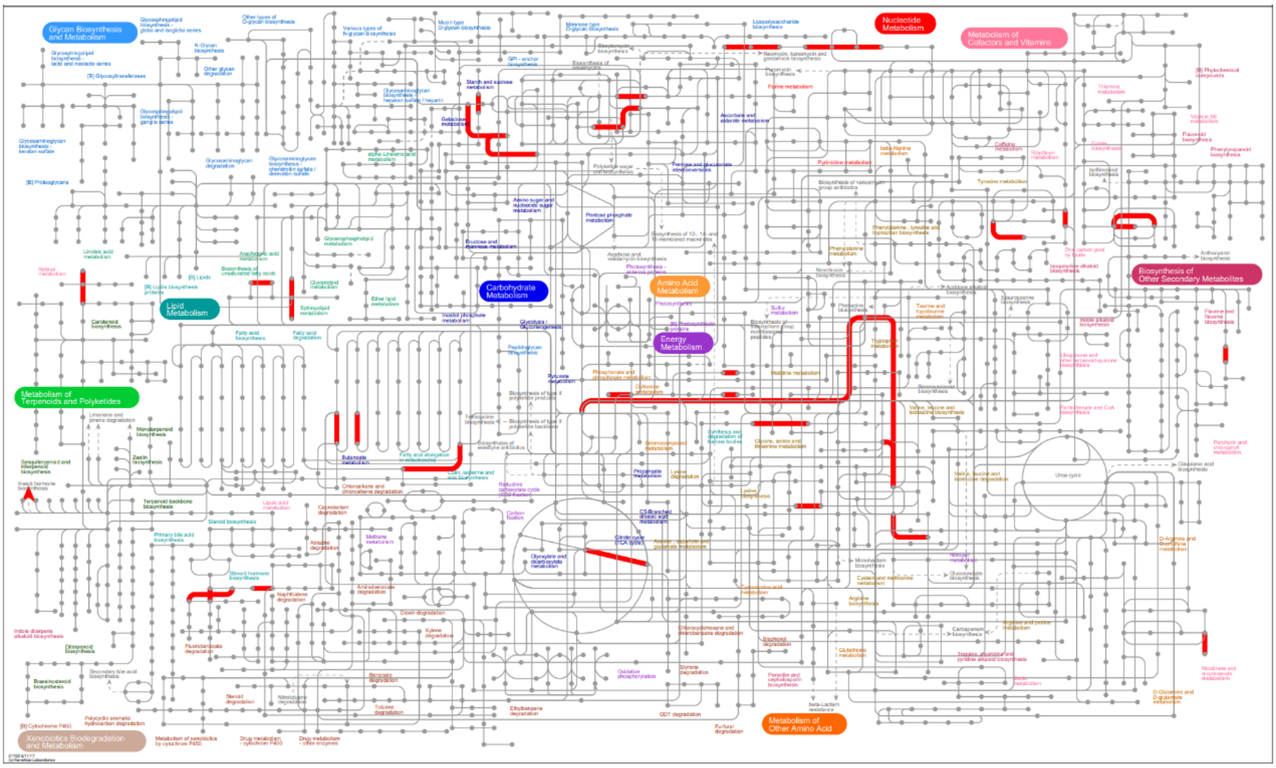

Figure S1. Analysis of RNA-seq of nematodes fed with different bacteria. IPath 3.0 software was used to visually analyze the metabolic pathways involved in these DEGs, and the metabolic information of the whole biological system was apparent. Most of the DEGs were enriched in energy metabolism.

Supplementary Material S3: GO and KEGG analysis of DEGs between worms fed p-AKK and p-OP50

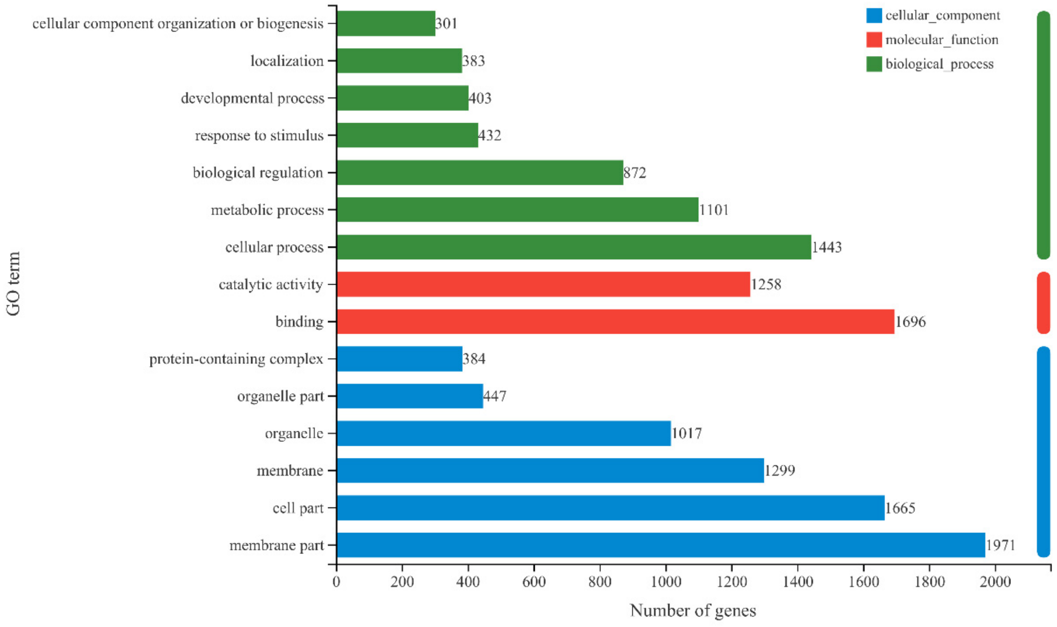

Figure S2. GO analysis of DEGs between worms fed p-AKK and p-OP50 in three main categories.

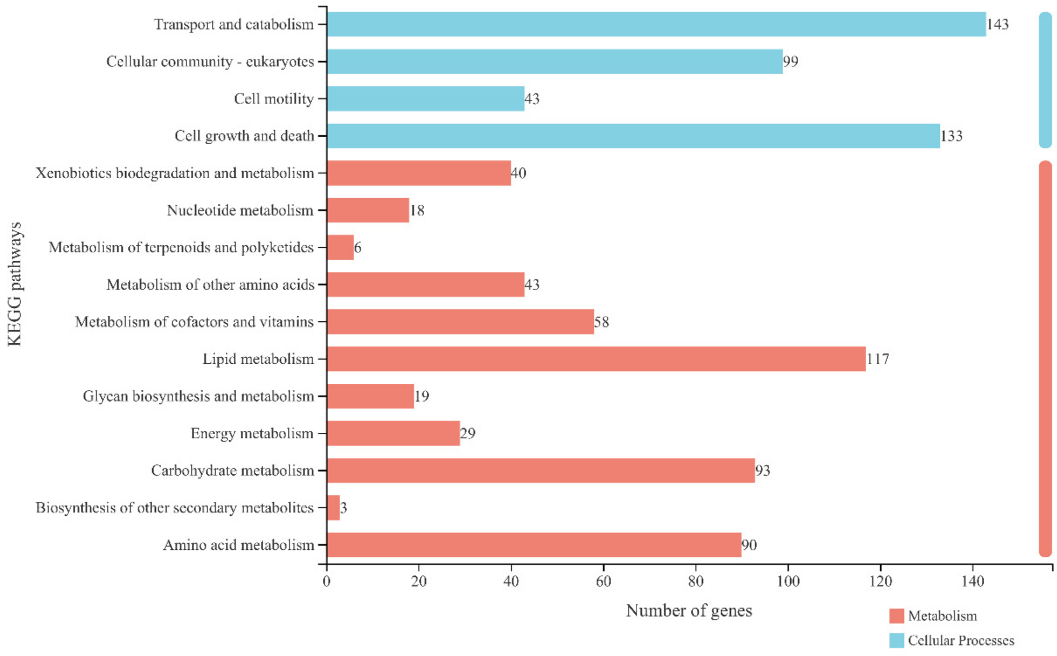

Figure S3. Analysis of DGEs of worms treated with p-AKK and p-OP50 by KEGG pathway classification.

Supplementary Material S4: KEGG Pathway

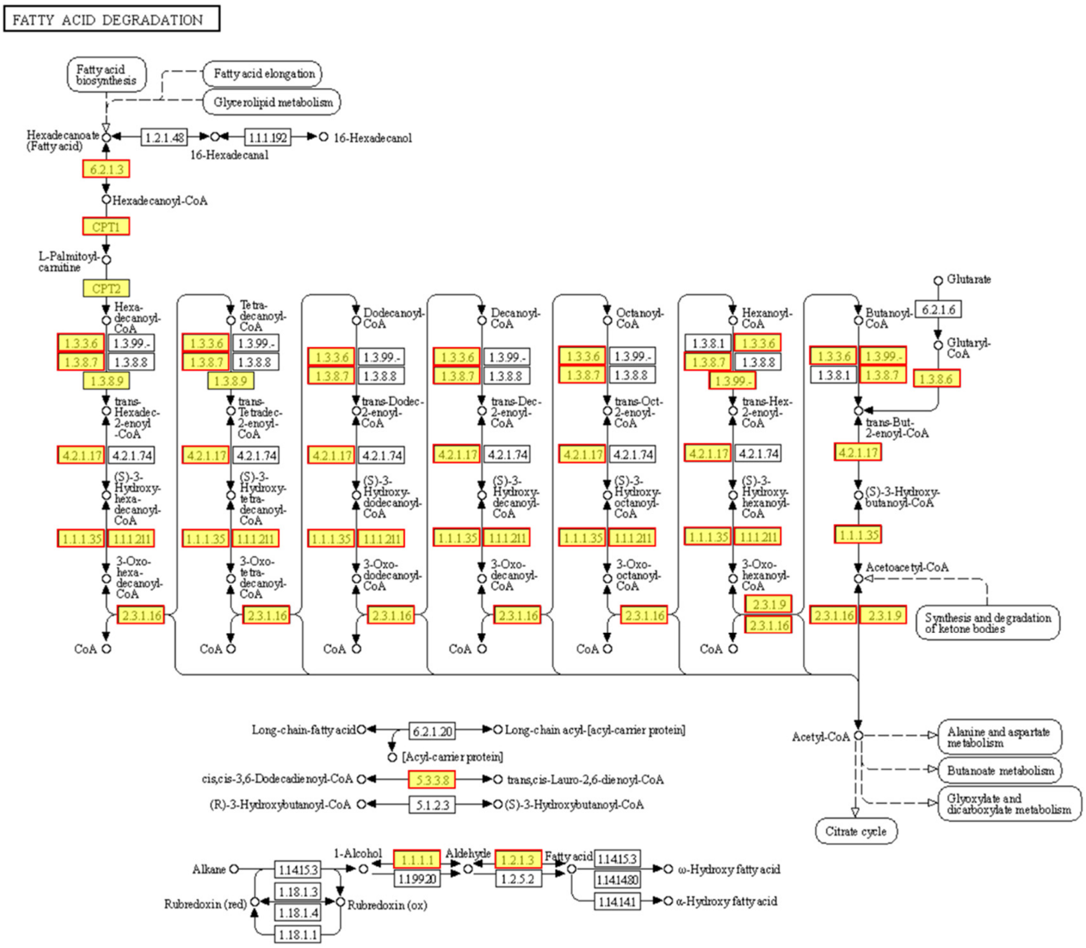

Figure S4. The effect of p-AKK on fatty acid degradation in KEGG pathway.

Table S2. The DEGs of fatty acid degradation pathway in nematodes mediated by p-AKK.

EC Number	Gene	Gene ID	Description	Status
6.2.1.3	acs-1	WBGene00018488	Fatty Acid CoA Synthetase	Down
	acs-2	WBGene00009221	Fatty Acid CoA Synthetase	Up
	acs-23	WBGene00011174	Fatty Acid CoA Synthetase	Up
	acs-15	WBGene00019920	Fatty Acid CoA Synthetase	Up
	acs-16	WBGene00018579	Fatty Acid CoA Synthetase	Up
	acs-18	WBGene00011173	Fatty Acid CoA Synthetase	Up
CPT 1	cpt-4	WBGene00019644	Carnitine Palmitoyl Transferase	Up
1.3.3.6	acox-1.2	WBGene00008565	Acyl-coenzyme A oxidase	Down
	acox-3	WBGene00019060	Acyl-coenzyme A oxidase	Up
	acox-1.6	WBGene00010336	Acyl-coenzyme A oxidase	Up
1.3.8.7	acdh-8	WBGene00019406	Acyl CoA DeHydrogenase	Up
4.2.1.17	ech-6	WBGene00001155	Enoyl-CoA Hydratase	Down
	ech-7	WBGene00001156	Enoyl-CoA Hydratase	Down
1.1.1.35	ech-9	WBGene00001158	Enoyl-CoA Hydratase	Down
1.1.1.211	ech-1.1	WBGene00001150	Enoyl-CoA Hydratase	Down
2.3.1.16	acaa-2	WBGene00009952	ACetyl-CoA Acyltransferase 2 homolog	Down
1.3.99.-	acdh-1	WBGene00016943	Acyl CoA DeHydrogenase	Down
	acdh-2	WBGene00015894	Acyl CoA DeHydrogenase	Down
	acdh-4	WBGene00020419	Acyl CoA DeHydrogenase	Up
2.3.1.9	kat-1	WBGene00002183	Acetyl-CoA acetyltransferase homolog	Down
1.3.8.6	F54D5.24	WBGene00271798	Unclarified	Up
1.1.1.1	sodh-1	WBGene00010790	Alcohol dehydrogenase 1	Up
1.2.1.3	alh-5	WBGene00000107	ALdehyde deHydrogenase	Up

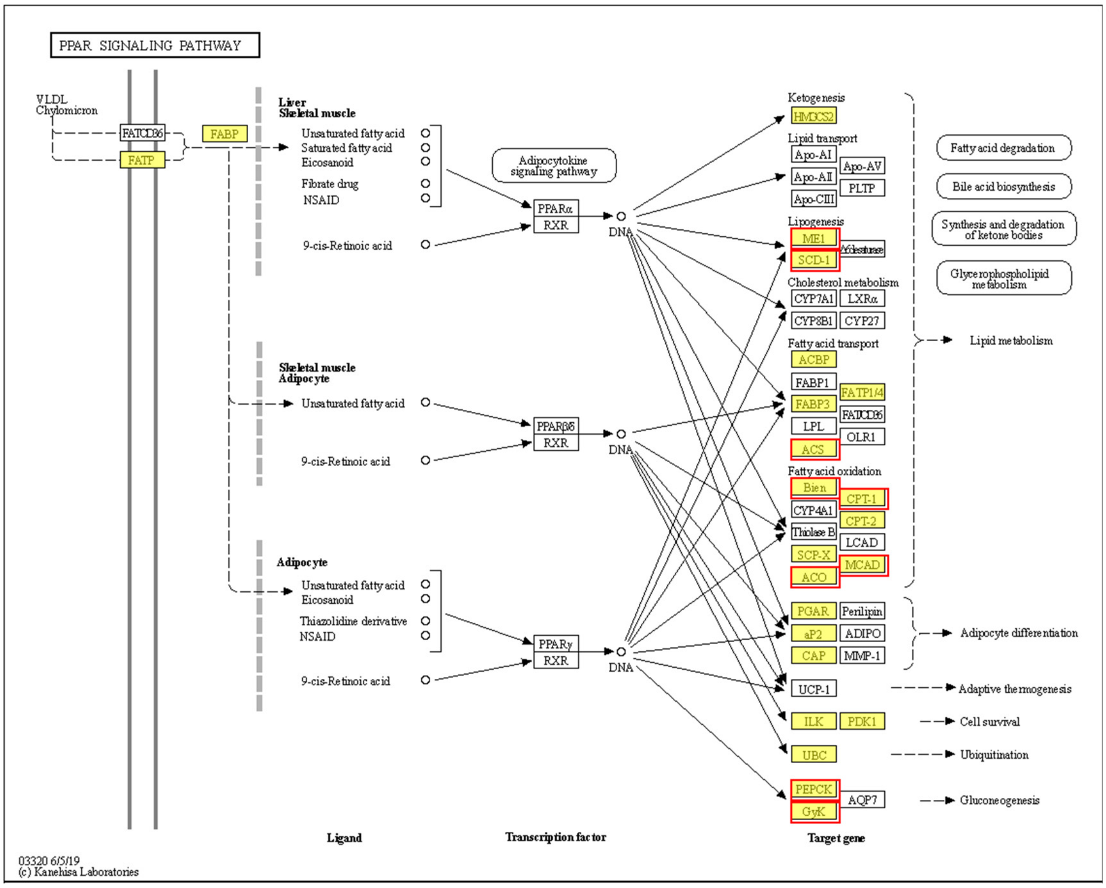

Figure S5. The effect of p-AKK on fat metabolism in PPAR pathway.

Table S3. The DEGs of PPAR pathway in nematodes mediated by p-AKK.

	Gene	Gene ID	Description	Function	Status
ME1	men-1	WBGene00012983	Malic enzyme	Lipogenesis	Down
SCD-1	fat-7	WBGene00001399	Delta(9)-fatty-acid desaturase	Lipogenesis	Down
ACS	acs-2	WBGene00009221	Fatty Acid CoA Synthetase	Fatty acid transport	Up
	acs-18	WBGene00011173	Fatty Acid CoA Synthetase	Fatty acid transport	Up
	acs-15	WBGene00019920	Fatty Acid CoA Synthetase	Fatty acid transport	Up
	acs-16	WBGene00019920	Fatty Acid CoA Synthetase	Fatty acid transport	Up
Bien	ech-9	WBGene00001158	Enoyl-CoA Hydratase	Fatty acid oxidative	Down
CPT-1	cpt-4	WBGene00019644	Carnitine Palmitoyl Transferase	Fatty acid oxidative	Up
MCAD	acdh-8	WBGene00019406	Acyl CoA DeHydrogenase	Fatty acid oxidative	Up
ACO	acox-3	WBGene00019060	Acyl-coenzyme A oxidase	Fatty acid oxidative	Up
	acox-1.5	WBGene00008167	peroxisomal acyl-coenzyme A oxidase 1.5	Fatty acid oxidative	Down
	acox-1.6	WBGene00010336	Acyl-coenzyme A oxidase	Fatty acid oxidative	Up
PEPCK	pck-3	WBGene00019151	Phosphoenolypyruvate Carboxy Kinase	Gluconeogenesis	Up
GYK	R11F4.1	WBGene00020007	glycerol kinase	Gluconeogenesis	Up

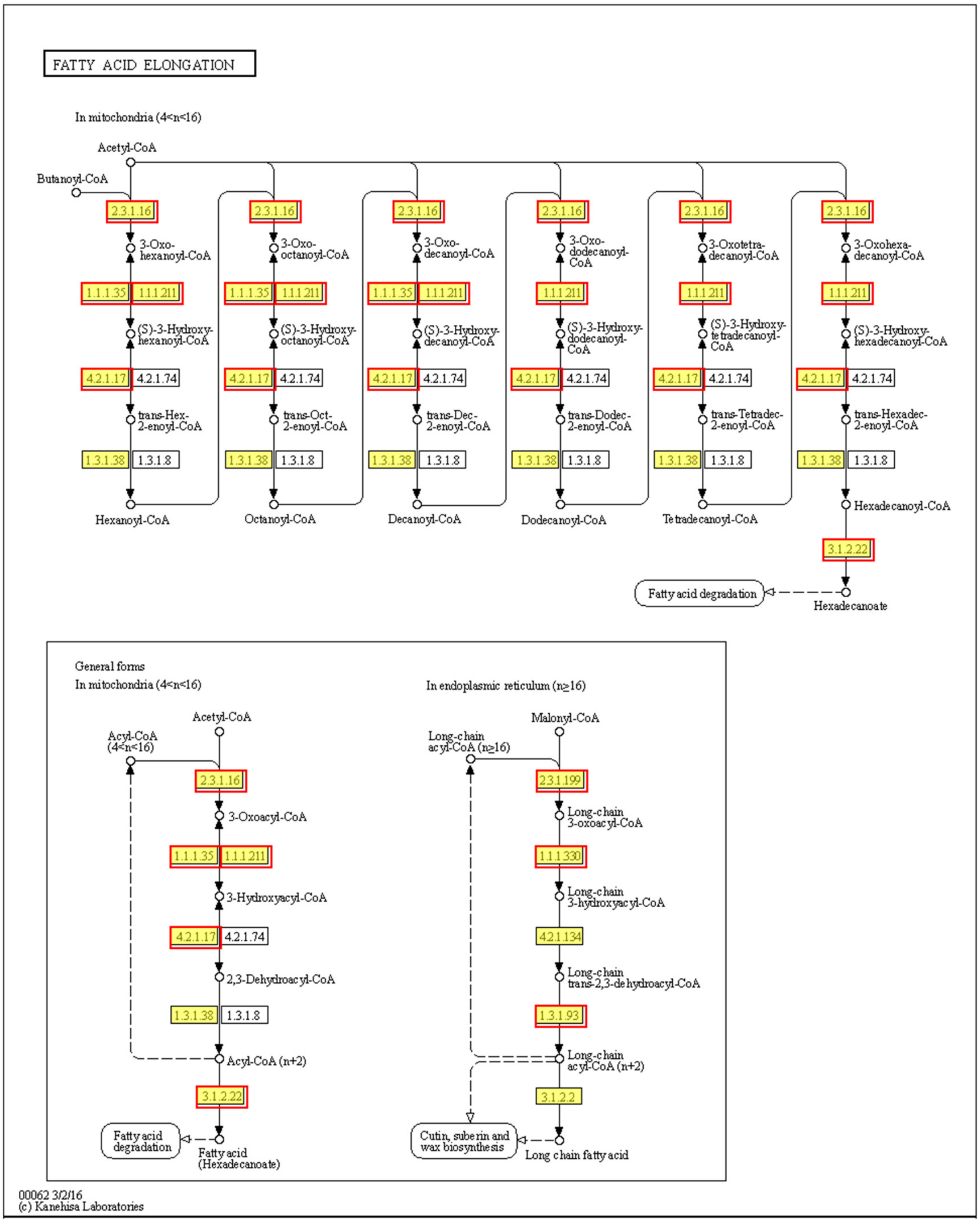

Figure S6. The effect of p-AKK on fatty acid prolongation in KEGG pathway.

Table S4. The DEGs of fatty acid prolongation pathway in nematodes mediated by p-AKK.

EC Number	Gene	Gene ID	Description	Status
2.3.1.16	acaa-2	WBGene00009952	ACetyl-CoA Acyltransferase 2	Down
1.1.1.35	ech-9	WBGene00001158	Enoyl-CoA Hydratase	Down
1.1.1.211	ech-1.1	WBGene00001150	Enoyl-CoA Hydratase	Down
4.2.1.17	ech-7	WBGene00001156	Enoyl-CoA Hydratase	Down
	ech-6	WBGene00001155	Enoyl-CoA Hydratase	Down
3.1.1.22	ppt-1	WBGene00004092	Palmitoyl-protein thioesterase 1	Up
23.1.199	elo-5	WBGene00001243	Elongation of very long chain fatty acids protein 5	Down
	elo-9	WBGene00001247	Elongation of very long chain fatty acids protein	Down
	elo-8	WBGene00001246	Elongation of very long chain fatty acids protein	Up
	elo-3	WBGene00001241	Putative fatty acid elongation protein 3	Down
	elo-2	WBGene00001240	Elongation of very long chain fatty acids protein	Down
	elo-7	WBGene00001245	Elongation of very long chain fatty acids protein	Up
1.1.1.330	rpn-13	WBGene00016981	Proteasomal ubiquitin receptor	Down
	dhs-27	WBGene00000990	oxidoreductase	Up
1.3.1.93	art-1	WBGene00000198	very-long-chain enoyl-CoA reductase	Down

Supplementary Material S5: DEGs and mRNA levels in fat metabolism of worms treated with p-AKK and p-OP50

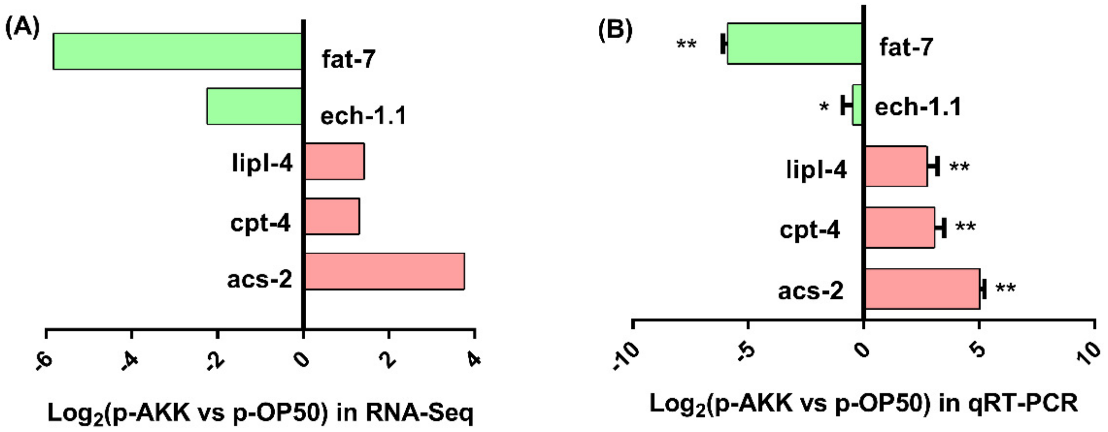

Figure S7. DEGs and mRNA levels in fat metabolism of worms treated with p-AKK and p-OP50 in RNA-Seq (A) and qRT-PCR (B).
